# Risk-Oriented Product Assembly System Health Modeling and Predictive Maintenance Strategy

**DOI:** 10.3390/s19092086

**Published:** 2019-05-05

**Authors:** Fengdi Liu, Yihai He, Yixiao Zhao, Anqi Zhang, Di Zhou

**Affiliations:** School of Reliability and Systems Engineering, Beihang University, Beijing 100083, China; fengdiliu@buaa.edu.cn (F.L.); zhyixiao@buaa.edu.cn (Y.Z.); zhanganqi0811@163.com (A.Z.); zhoudimail@163.com (D.Z.)

**Keywords:** assembly system, health risk, key reliability characteristics (KRCs), predictive maintenance, assembled product reliability

## Abstract

Assembly quality is the barometer of assembly system health, and a healthy assembly system is an important physical guarantee for producing reliable products. Therefore, for ensuring the high reliability of products, the operational data of the assembly system should be analyzed to manage health states. Therefore, based on the operational data of the assembly system collected by intelligent sensors, from the perspective of quality control based on risk thinking, a risk-oriented health assessment method and predictive maintenance strategy for managing assembly system health are proposed. First, considering the loss of product reliability, the concept of assembly system health risk is proposed, and the risk formation mechanism is expounded. Second, the process variation data of key reliability characteristics (KRCs) collected by different sensors are used to measure and assess the health risk of the running assembly system to evaluate the health state. Third, the assembly system health risk is used as the maintenance threshold, the predictive maintenance decision model is established, and the optimal maintenance strategy is determined through stepwise optimization. Finally, the case study verifies the effectiveness and superiority of the proposed method. Results show that the proposed method saves 37.40% in costs compared with the traditional method.

## 1. Introduction

The assembly process is a classic and critical step in manufacturing, whose quality considerably affects product reliability [1,2]. The assembly system is a direct carrier of the assembly process, and its health is an important factor affecting the assembly quality [3]. Therefore, health management of the assembly system to ensure its healthy operation is a major means for improving product reliability. Hence, manufacturers must always pay attention to the health of the assembly system. To analyze the health state of the assembly system, it is necessary to obtain the real-time operational data. In the context of intelligent manufacturing, various types of smart sensors are used to collect process data to establish a data foundation [4]. Since device anomalies can propagate between sensors and devices through potential connections between sensors [5], real-time system health can be understood through the sensor data foundation to predict trends in device failures. In this manner, manufacturers can plan timely maintenance and improve the assembly quality and reliability of the final product [6].

The new version of the quality management standard ISO 9001:2015 emphasizes the risk-based thinking for product quality management; as such, risk-based thinking becomes an inevitable trend of quality analysis [7,8]. Therefore, risk-based thinking is considered to manage the decline of assembly system health, and the concept of assembly system health risk is proposed. The system health decline initially leads to a decline in the assembly process quality and then in product reliability; therefore, in essence, health risk is a type of quality risk. In actual production, when the assembly system health is low, process variations occur, thereby greatly affecting the quality of the assembly process. In this context, Thornton [9,10] used risk-based thinking to propose a variation risk management (VRM) theory, which was applied to product assembly quality management. Thornton emphasized that, in the assembly process, if the measures are not fully considered or adopted to quantify and control the process variation effectively, then the resulting quality risk would considerably affect product reliability. Montgomery [11] and Kaya and Özer [12] defined quality risk as the risk to manufacturer and user caused by classification errors in quality testing. Lee [13] believed that the processing quality cost can directly represent the quality risk. Moreover, a general processing loss-based process capability index was constructed on the basis of expectation and Taguchi loss functions to describe the quality risk under symmetric or asymmetric tolerance conditions, and the conversion method for the index and product failure rate was proposed. Debón and Garcia-Díaz [14] linked the failure rate to the product quality risk and defined the defective rate of finished products as the main indicator of quality risk; they achieved a further accurate prediction of the coil batch risk by monitoring the key process risk characteristics. Park and Shrivastava [15] emphasized that the traditional assembly ideas that only pay attention to the assembly quality state and disregard the impact of assembly quality decline on the stage of use of the finished product are immature, and these ideas neither propose the concept of risk management quality analysis nor use risk-based thinking to explain the quality of product assembly. On this basis, He et al. [16] targeted the general manufacturing process by considering the cumulative effect of process variation; they focused on the qualification rate of the key manufacturing end to characterize the batch quality risk. Cui et al. [17] considered the effect of assembly process variation on product reliability reduction and quality accident risk; they defined product assembly quality risk and then modeled and analyzed the risk on the basis of the QRR chain (That is the chain structure explaining the relationship between assembly process quality Q_A_, product reliability R_P_, and failure accident risk R_FA_.). 

However, only the risk analysis was completed in the abovementioned studies. The system health states were not evaluated in accordance with the risk level, and the equipment maintenance strategy was not determined. At present, with the continuous advancement of technology, predictive maintenance [18] has become the representative of advanced maintenance technology. Predictive maintenance improves the shortcomings of under- or over-maintenance that may result from scheduled maintenance. Predictive maintenance can forecast the trend of failures based on equipment fault diagnosis and health states and develop maintenance strategies and proactive maintenance. Therefore, it is also called condition-based maintenance. Fault diagnosis is important for predictive maintenance. Targeting the ground-source heat pump (GSHP) system, Cai et al. [19] proposed a fault diagnosis approach based on multi-source information fusion Bayesian network to improve the accuracy of fault diagnosis. Oriented by data drive, a fault diagnosis method based on Bayesian network is proposed for the three-phase inverter of the PMSM (permanent magnet synchronous motor) drive system [20]. Cai et al. [21] performed a fault diagnosis on the complex electronic systems. A method based on dynamic Bayesian network (DBN) to identify component faults was proposed, and the purpose of distinguishing fault types was achieved. For predictive maintenance, Zhou [22] proposed failure rate growth and age reduction factors based on the degradation caused by the incomplete maintenance of equipment; he established a predictive maintenance model centered on system reliability. The modular mode is widely used in the field of predictive maintenance. Deloux et al. [23] proposed a predictive maintenance method that considers statistical process control for two failure modes, namely, excessive degradation and shock. By considering the maintenance opportunity and cost, Li et al. [24] proposed a predictive maintenance strategy for a series-parallel assembly system based on equipment state detection information to ensure the healthy operation of the equipment. For marine oil separation systems, Marichal et al. [25] combine predictive maintenance with genetic neuro-fuzzy system to determine initial failures. Shi and Zeng [26] established a predictive maintenance model by considering the real-time residual life and maintenance cost of the system for dynamic opportunistic multi-station systems. He et al. [27] proposed an integrated predictive maintenance strategy that combines product quality control and mission reliability constraints regarding the intelligent manufacturing philosophy of ‘prediction and manufacturing’. Wu et al. [28] pointed out the importance of predictive maintenance and prognostics health management (PHM) in the field of cyber-physical systems (CPS), and optimized the deep learning model for fault diagnosis. Gilardoni et al. [29] used a data-based system health state prediction method to establish a predictive maintenance model for the equipment based on data mining. However, in current models for predictive maintenance, risk-oriented system health is rarely used as a maintenance threshold to develop the specific maintenance strategy. Therefore, this study proposes a risk-oriented health assessment model of product assembly system and a predictive maintenance strategy by considering the effect of assembly system health decline on product reliability. The goal is to improve the health of assembly systems for enhancing product reliability through maintenance. The main contributions are as follows:

(1) To analyze the health states of the product assembly system quantitatively, the concept of assembly system health risk is proposed, and the risk connotation and formation mechanism are expounded, and the risk analysis data foundation is established based on big operational data of assembly system collected by smart sensors.
(2) The concept of key reliability characteristics (KRC) is proposed. The quantitative relationship between the assembly process quality of assembled products and the assembly system health risk is established by KRC variations.
(3) With the assembly system health risk as the threshold and the minimum total cost as the goal, the predictive maintenance decision-making model is established, and the optimal predictive maintenance strategy of the product assembly system is obtained.

The remainder of this paper is organized as follows: In Section 2, the connotation and formation mechanism of assembly system health risk and health risk analysis data foundation are expounded. In Section 3, the concept of KRC is proposed, and a quantitative relationship among the KRC assembly process variations and the health risk of the product assembly system is established, and the assembly system health assessment is completed. In Section 4, the health risk of the product assembly system is used as the predictive maintenance threshold, and a maintenance decision-making model is established to find the optimal maintenance strategy at the lowest total cost. In Section 5, a case study is provided. In Section 6, conclusions and directions for future research are provided.

## 2. Health Risk Analysis Basis of the Assembly System

### 2.1. Health Risk Connotation of the Assembly System

As the final step before the product is formed, the assembly process is performed by an assembly line consisting of various assembly equipment. Among them, the system consisting of all the equipment on the assembly line is called the assembly system. With the improvement of living standards, consumer expectation for product functions are gradually increasing, which also leads to the increasingly complex product structure, with characteristics of numerous parts, various mating modes, and high precision, and more complicated failure modes. Therefore, to ensure the high reliability of complex products, manufacturing companies have higher requirements for assembly systems, and it is also of great significance for assembly system health risk assessment and equipment maintenance strategies.

The manufacturing process refers to the stage of product manufacturing reliability formation. The assembly process, as the most classic and core part of the manufacturing stage, remarkably affects product manufacturing quality and reliability. Without considering human factors, such quality is determined by the assembly system health. Assembly quality refers to the quality of the assembly process, mainly in the quality state of the assembled product during the assembly process, such as the size of the variation, the number of manufacturing defects, etc. Therefore, the assembly system health is an important guarantee for product quality and reliability. Then, to transform quality management mode from passive adjustment to active improvement, risk-based thinking is used to analyze and assess assembly system health. Then, the assembly system health risk is proposed. That is, the risk of abnormity quality accidents due to the product reliability loss caused by the decline in the health state of the assembly system is the health risk of the product assembly system. Thus, the connotation of the assembly system health risk is the reliability loss of the product. In today’s highly automated assembly process, various types of intelligent sensors are used to collect the big operational data of the assembly system, and the collected data is analyzed. And a risk assessment can be conducted around the effect of risk on product reliability, and a maintenance strategy can be formulated to improve the product reliability for risk mitigation.

### 2.2. Formation Mechanism of Assembly System Health Risk

**Figure 1:**
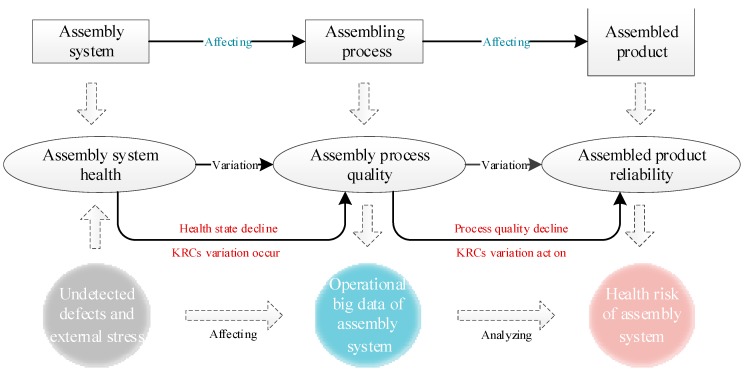
Formation mechanism of the assembly system health risk.

The risk formation mechanism must be clarified to reduce the assembly system health risk for improving the assembly system health and manufacturing reliability of the assembled product. The risk connotation mentioned in Section 2 helps explore the risk formation mechanism. On this basis, Figure 1 shows the specific formation mechanism of the assembly system health risk.

When the components of the product have been produced, the assembly process is entered. First, as shown in the left part of Figure 1, the reliabilities of each assembly equipment have different losses due to the internal undetected defects (e.g., locating pin wear and insufficient clamping accuracy) and external stress (e.g., environment stress). The degradation leads to a decrease in the assembly system health, which causes its operating state to decline. Second, as shown in the middle part of Figure 1, the poor operating state of the assembly system, as the carrier for the assembly process, can cause dimensional variations among the components during assembly, and the corresponding variation data can be captured by the sensors. This case results in a decline in the quality of the assembly process, which influences the product [30] and causes its reliability to further degrade, as shown.

(1) $$R_{d}-R_{m}=\Delta R_{p}+\Delta R_{a}$$

where $R_{d}$ represents the design reliability of the product, $R_{m}$ is the manufacturing reliability of the product, $\Delta R_{p}$ denotes the reliability loss of the product in the production process of components, and $\Delta R_{a}$ indicates the reliability loss of the product during assembly.

As this study focuses on the influence of the dimensional variations of the assembly process on product manufacturing reliability, the concept of KRCs is proposed as follows: The product characteristics which are closely related to the product manufacturing reliability are called the key reliability characteristics (KRCs), that is, KRCs are sensitive to product manufacturing reliability. Accordingly, the product characteristics that are insensitive to product manufacturing reliability are referred to as non-KRCs. Therefore, dimensional variations generated during assembly can be classified into two types, namely, dimensional variations of KRC and dimensional variations of non-KRC. KRCs have the property of product manufacturing reliability sensitivity. Hence, the manufacturing reliability loss of product caused by the assembly process variations of KRCs is substantial. Accordingly, the assembly process variations of non-KRCs have less effect on the manufacturing reliability of the product. Thus, $\Delta R_{a}$ can be expressed as follows:

(2) $$\Delta R_{a}=(\Delta R_{a}{)}_{KRC}+{\left(\Delta R_{a}\right)}_{nonKRC}$$

where $(\Delta R_{a}{)}_{KRC}$ and $(\Delta R_{a}{)}_{nonKRC}$ represent the reliability losses of the product caused by the assembly process variations of KRCs and non-KRCs, respectively.

Given that the value of dimensional variation is small, the dimensional variations of non-KRCs have a negligible effect on product reliability. By contrast, given the close relationship between KRCs and product reliability, its small dimensional variation can still substantially influence the product. Therefore, this study only considers the process dimensional variation of KRCs. Equation (2) can be rewritten as follows:

(3) $$\Delta R_{a}=(\Delta R_{a}{)}_{KRC}$$

Finally, as shown in the right part of Figure 1, the assembly process for product has the characteristics of multi-stations. Therefore, the process variation can continuously be transmitted to the downstream station as the multi-station assembly process progresses. If the KRC dimensional variations occur during assembly, these variations can continuously transmit to the downstream station, that causes variations accumulation. Then, the variations of KRCs can influence the product, thereby causing its manufacturing reliability loss. Then, the health risk of the product assembly system occurs. Due to the different health level of the assembly system, the process variations of the KRCs are also different, that results in different levels of health risk of the assembly system. Therefore, the assembly system health risk stems from the assembly system, forms during assembly, and appears in the assembled product. 

This study establishes a quantitative relationship between the assembly process variations of KRCs and the health risk of the product assembly system to obtain the risk level of the system. Then, to mitigate the risk and improve the reliability of product, the risk level is used to characterize the assembly system health and develop the predictive maintenance strategies for different health states. 

### 2.3. Health Risk Analysis Data Foundation Based on Smart Sensors

The assembly system is a collection of various devices. The operation process generates a large amount of operational data, which includes a quantity of information in various dimensions, such as equipment health state, maintenance, and risk management. If the real-time data of the assembly system can be collected, transmitted, fused, and analyzed, that can more accurately and timely grasp the equipment information and analyze the transmission and accumulation of health risk, which has far-reaching significance for improving the health of the system and improving the manufacturing reliability of the assembled products.

In the context of smart manufacturing, various types of smart sensors are widely used for data collection. With the data acquisition function of the sensor, when the assembly system starts running, the sensors located on each device can capture the real-time state data of the device. Due to the different types of sensors and the large number of devices, this type of data has the characteristics of diverse data types and rich information, which is called big operational data of the assembly system. According to the assembly system health risk analysis method, the acquired big operation data of the assembly system is screened, the data foundation is established. Then, the data foundation is analyzed to achieve the purpose of health risk analysis and equipment maintenance. And the specific analysis method is described in Section 3. 

## 3. Health Risk Modeling of Assembly System

**Figure 2:**
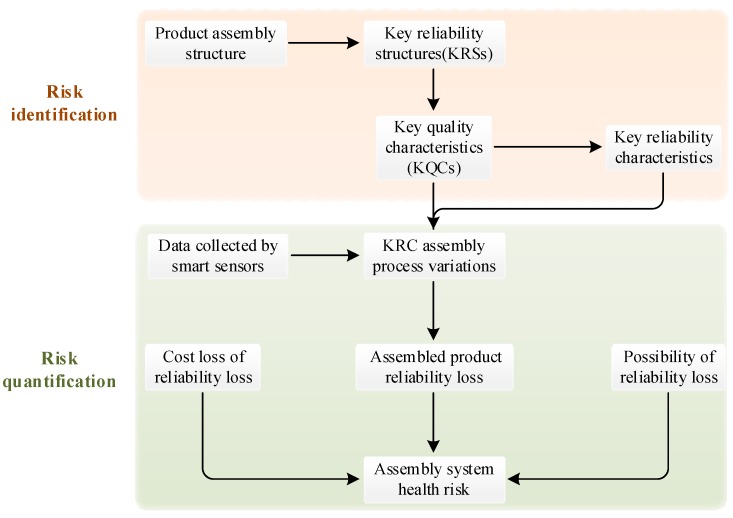
Framework of the health risk assessment model for the product assembly system.

Based on the formation mechanism of the assembly system health risk, the decline in system health is the root cause of the risk, and the assembly process variations of the KRCs are the direct causes of the risk. Therefore, modeling the quantitative relationship between product KRC variations and the assembly system health risk is the most important part for assessing the assembly system health. Thornton [7,8] proposed the VRM model for the risk caused by process variation. On this basis, a health risk assessment model for the product assembly system is proposed. Figure 2 shows the framework of this model.

The risk assessment framework is divided into three steps: risk identification, risk quantification, and risk classification. In the following sections, each step is specifically expounded.

This section may be divided by subheadings. It should provide a concise and precise description of the experimental results, their interpretation as well as the experimental conclusions that can be drawn.

### 3.1. Risk Identification

The process variations of KRCs are the direct causes of the assembly system health risks. Therefore, the KRCs should be determined during assembly for the risk analysis. The potential defects of a static assembly system are difficult to determine. Nonetheless, when the system is operating, the variations of KRCs can be found during assembly. Moreover, based on the analysis of the variation result, the hidden defects of the system can be obtained and repaired. Therefore, as the basis of risk analysis and classification and risk mitigation, the risk identification stage should focus on identifying KRCs.

**Figure 3:**
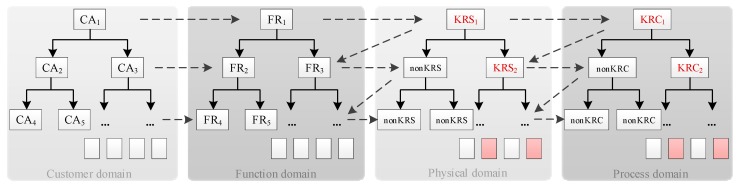
Key reliability characteristic (KRC) identification structure diagram based on axiomatic domain mapping.

The KRC identification includes the following four steps:

- Step 1. Determining the product assembly structure. The product is decomposed from top to bottom in accordance with the design requirements to determine the product assembly structure, which comprises product assembly, sub-assembly, components, parts, and other structural details, during assembly.
- Step 2. Determining the key reliability structures (KRSs). Based on product function and structure, the sensitivity requirements of product structure are determined. On this basis, the product structures whose variations are sensitive to the product reliability are screened out. The structures that are screened out are called the KRSs.
- Step 3. Determining the KRCs. Based on the identified KRSs information of product, the corresponding process parameters are identified by axiomatic domain mapping to determine the assembly process KRCs, as shown in Figure 3.
- Step 4. Updating the KRCs database. In the context of rapid product upgrades, the product KRC database is updated regularly based on the results of the KRC identification to analyze the health risk of the product assembly system in a timely and effective manner.

where, CA refers to customer attribute and FR refers to functional requirement.

### 3.2. Risk Quantification

After the risk identification, the key indicator of KRCs are obtained. Subsequently, to analyze the risk, the process data needs to be processed. The smart sensors located on the assembly system collect a large amount of process data, which can be filtered by the identified key indicator types to form the big operational data of the assembly system. Based on the operational big data of the assembly system, the real-time assembly variations of KRCs are obtained, and the influence of the assembly dimension variations of KRCs on product manufacturing reliability is quantified to assess the assembly system health risk. The specific steps are as follows:

Three parameters are proposed to describe and quantify risk. Parameter L represents the product reliability loss caused by the KRC assembly process variations in the assembly stage. Parameter $C_{RH}$ denotes the loss cost caused by the product reliability loss brought about by KRC assembly process variations. Parameter P indicates the probability of corresponding KRC assembly process variations. Correspondingly, the total risk RH can be expressed as follows:

(4) $$RH=L\times C_{RH}\times P$$

The value of parameter L is quantified by the effect of the KRC assembly process variation on the assembled product reliability loss. The assembly process variation of the *i*th KRC can be expressed as follows:

(5) $$\Delta X_{i}=\frac{\partial X_{i}}{\partial x_{i1}}\Delta x_{i1}+\frac{\partial X_{i}}{\partial x_{i2}}\Delta x_{i2}+\ldots +\frac{\partial X_{i}}{\partial x_{in}}\Delta x_{in}$$

where, $X_{i}$ is the assembly process variation of the *i*th KRC, and $\Delta X_{i}$ represents the amount of change in $X_{i}$, $x_{ik}$ denotes the assembly process variation of the *i*th KRC on the *k*th station, $\frac{\partial X_{i}}{\partial x_{ik}}$ refers to the partial derivative of $X_{i}$ with respect to $x_{ik}$.

As the variation cumulative quantitative model of the KRC is subject to the linear model, hence $\frac{\partial X_{i}}{\partial x_{ik}}=1$. Then, Equation (5) can be simplified as

(6) $$\Delta X_{i}=\Delta x_{i1}+\Delta x_{i2}+\ldots +\Delta x_{in}$$

where

(7) $$x_{ik}=x_{ik1}{}^{T}+x_{ik2}{}^{T}+\ldots +x_{ikn}{}^{T}$$

where $x_{ikj}$ is the assembly process variation of the *i*th KRC on the *k*th station on the *j*th positioning pin.

After the individual KRC variations are quantified, the link is established between the KRC variations and product reliability loss. The effect of KRC variations on the product reliability is subject to the linear model.

(8) $$\Delta L=\frac{\partial L}{\partial X_{1}}\Delta X_{1}+\frac{\partial L}{\partial X_{2}}\Delta X_{2}+\ldots +\frac{\partial L}{\partial X_{n}}\Delta X_{n}+o$$

where o is the high-order infinitesimal, which can be ignored when $\Delta X_{i}$ is small. In the actual assembly process, the process variations are small. Therefore, Equation (8) can be simplified as

(9) $$\Delta L=\sum_{i=1}^{n}K_{i}\Delta X_{i}+D$$

where $K_{i}$ denotes a constant coefficient matrix and D is constant, which are determined by engineering experience and the fuzzy TOPSIS method [31].

Since different levels of health risk have different occurrence probabilities and result in different costs of quality accident, the product assembly system health risks should be graded to determine the cost and probability. The rating is based on the following:

(10) $$k=\frac{L}{L_{\max}}$$

where k is the reliability loss level coefficient, in which the loss is small when 0<k≤0.4, the loss is at a medium level when 0.4<k≤0.75, and the loss is substantial when 0.75<k≤1. As this study only discusses the KRC variations of the qualified products during assembly, the unqualified products are not considered. Therefore, k cannot be greater than 1. $L_{\max}$ is the maximum value of the assembled product manufacturing reliability loss.

Based on the value of k, the values of parameters $C_{RH}$ and P can be obtained by referring to the table of risk parameters which can be obtained from the manufacturer.

Finally, the parameters are brought to Equation (4) to obtain the value of the assembly system health risk.

## 4. Health Risk-Oriented Predictive Maintenance Strategy for the Assembly System

### 4.1. Health Risk-Oriented Predictive Maintenance Mechanism 

**Figure 4:**
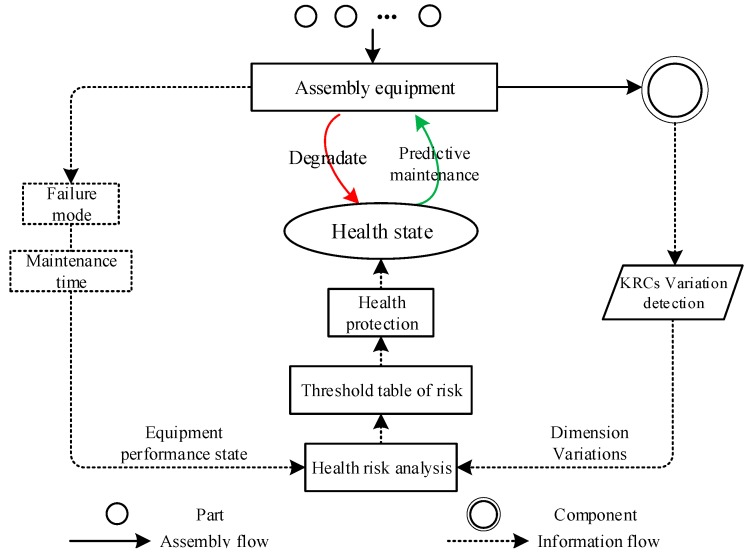
Schematic of the predictive maintenance mechanism of the equipment layer.

To improve the manufacturing reliability of products and reduce quality accidents, the maintenance means should be used to improve the assembly system health. Figure 4 shows the schematic of the predictive maintenance decision-making mechanism of the assembly equipment.

During the operation of the assembly system, different levels of system health risks reflect the various performance states. Combined with the characteristics of equipment performance polymorphism, and the performance degradation state of the system characterized by the health state of the assembly system, the performance state is affected by parameters, such as system health risk value and maintenance time. Generally, under certain conditions, the maintenance time of each failure mode of the equipment is constant. Therefore, based on the health risk modeling and analysis of the assembly system in Section 3, the preventive maintenance decision is made for the equipment operating state with the health risk as the threshold and the minimum cost as the goal.

Specifically, the health risk indicator that characterizes the operating state of the assembly system is used as a threshold to perform predictive maintenance activities. By analyzing the total cost (e.g., maintenance and product reliability degradation costs) under different health risk thresholds, the optimal predictive maintenance threshold of the assembly system is determined based on the minimum total cost. When the threshold is reached, the corresponding maintenance activities are performed. The maintenance process is used to improve the performance and health state of the assembly system; it is not utilized to restore the performance to as new but to reset the process controllable variables to the design nominal values. 

### 4.2. Health Risk-Oriented Predictive Maintenance Decision-Making Model

**Figure 5:**
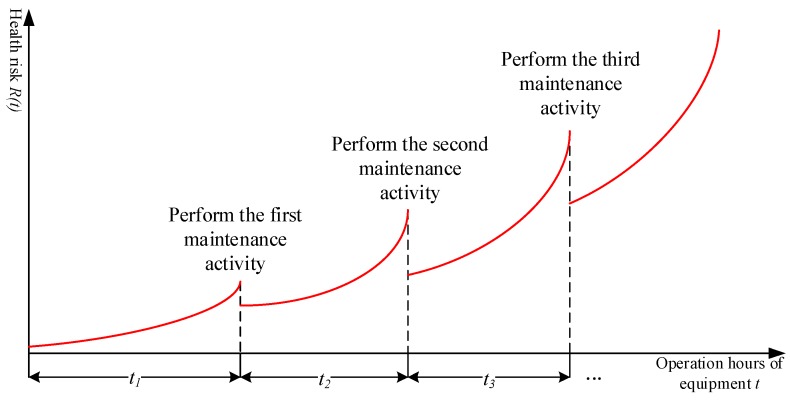
Chart of health risk change trend under maintenance activities.

Health risk is a barometer of assembly system performance degradation. Although predictive maintenance activities can improve performance and reduce assembly system health risk, completely eliminating the risk is impossible. Therefore, the risk escalation factor β is introduced to describe the trend of health risk after predictive maintenance, and Figure 5 shows the evolution model of health risk during the progress of predictive maintenance activities.

After the implementation of the *m* + 1th maintenance activity, the assembly system health risk is reduced, but it is still greater than the risk value after the implementation of the *m*th maintenance activity. Therefore, as the service life of the equipment increases, the risk shows an upward trend. Nonetheless, predictive maintenance strategies can limit the mitigation of risk increases.

Based on the predictive maintenance decision-making process shown in Figure 4, to guarantee the cost-effectiveness ratio, the related expenses that need to be considered in the predictive maintenance decision-making are composed of three parts: $c_{M}$, $c_{A}$, and $c_{R}$.

(11) $$C=c_{M}+c_{A}+c_{R}$$

where C is the total cost, $c_{M}$ denotes the maintenance cost, $c_{A}$ represents the cost of assembly capacity loss, and $c_{R}$ indicates the cost of the manufacturing reliability loss of the assembled product. These costs can be characterized by quantitative means as follows:

#### 4.2.1. Maintenance Cost

When the health state of the assembly system declines and the risk reaches the preset maintenance threshold, predictive maintenance will be performed. Assuming that the cost of each maintenance activity is the same and unchanged, the maintenance cost depends on the number of maintenance activities performed in the maintenance period t. Thus, the maintenance cost in the maintenance period is expressed by Equation (12).

(12) $$c_{M}=Nc_{e}$$

where $c_{e}$ is the cost of each maintenance and N indicates the number of maintenance activities performed in the maintenance period t.

(13) $$N=\frac{\xi {\left(RH_{\max}-RH_{T}\right)}^{2}}{\Delta RH}$$

where ξ is the risk reduction factor, $RH_{\max}$ represents the maximum health risk value allowed by the risk requirements of the manufacturer, $RH_{T}$ denotes the health risk threshold, and ΔRH indicates the amount of reduced assembly system health risk after performing maintenance activities.

Then, Equation (12) can be further expressed as follows:

(14) $$c_{M}=\frac{\xi }{\Delta RH}c_{e}{\left(RH_{T}\right)}^{2}-2\frac{\xi }{\Delta RH}c_{e}RH_{\max}RH_{T}+\frac{\xi }{\Delta RH}c_{e}{\left(RH_{\max}\right)}^{2}$$

#### 4.2.2. Cost of Assembly Capacity Loss

During the operation of the assembly system, equipment shutdown will interrupt the assembly activities, resulting in efficiency loss in addition to increased maintenance cost, that is, the assembly cycle becomes long due to equipment shutdown. As such, producing normal benefits is impossible. Therefore, the cost of assembly capacity loss depends on the equipment downtime in the assembly mission planning period, as shown in the following equation:

(15) $$c_{A}=\delta \left(\sum_{m=1}^{N}\int_{0}^{t_{m}}\beta dt\right)$$

where δ is the cost corresponding to the loss of unit assembly capacity and $t_{m}$ denotes the downtime of each maintenance activity. Assume that the equipment has only one failure mode, then

(16) $$t_{1}=t_{2}=\ldots =t_{m}$$

Then, Equation (15) can be further expressed as follows:

(17) $$c_{A}=\frac{\zeta \delta t_{m}\beta }{\Delta RH}{\left(RH_{T}\right)}^{2}-\frac{2\zeta \delta t_{m}\beta RH_{\max}}{\Delta RH}RH_{T}+\frac{\zeta \delta t_{m}\beta }{\Delta RH}{\left(RH_{\max}\right)}^{2}$$

#### 4.2.3. Cost of Manufacturing Reliability Loss of the Assembled Product

On the basis of Section 2, the process variations of the KRCs during assembly leads to the manufacturing reliability loss of the assembled product, resulting in increased cost of reliability loss. The KRCs of the output products of each equipment are assumed to be 100% tested. As the KRC variations are the main causes of reliability loss and the two are positively correlated, the calculation process of $c_{R}$ is shown as follows:

(18) $$c_{R}=\sigma L$$

where σ is the cost corresponding to unit reliability loss.

Based on the relationship between L and RH in Section 3.2, Equation (18) can be further expressed as follows:

(19) $$c_{R}=\sigma \frac{RH}{C_{RH}\times P}$$

Finally, based on the cost-effectiveness ratio, by comprehensively considering the total cost and system health risk, the maintenance decision is made, and the predictive maintenance method is determined. Specifically, the health risk that characterizes the health states of the assembly system is used as the maintenance threshold. By analyzing the total cost under different health risk thresholds, the maintenance strategy is determined based on the minimum total cost. The following figure shows the iterative numerical optimization flow chart.

**Figure 6:**
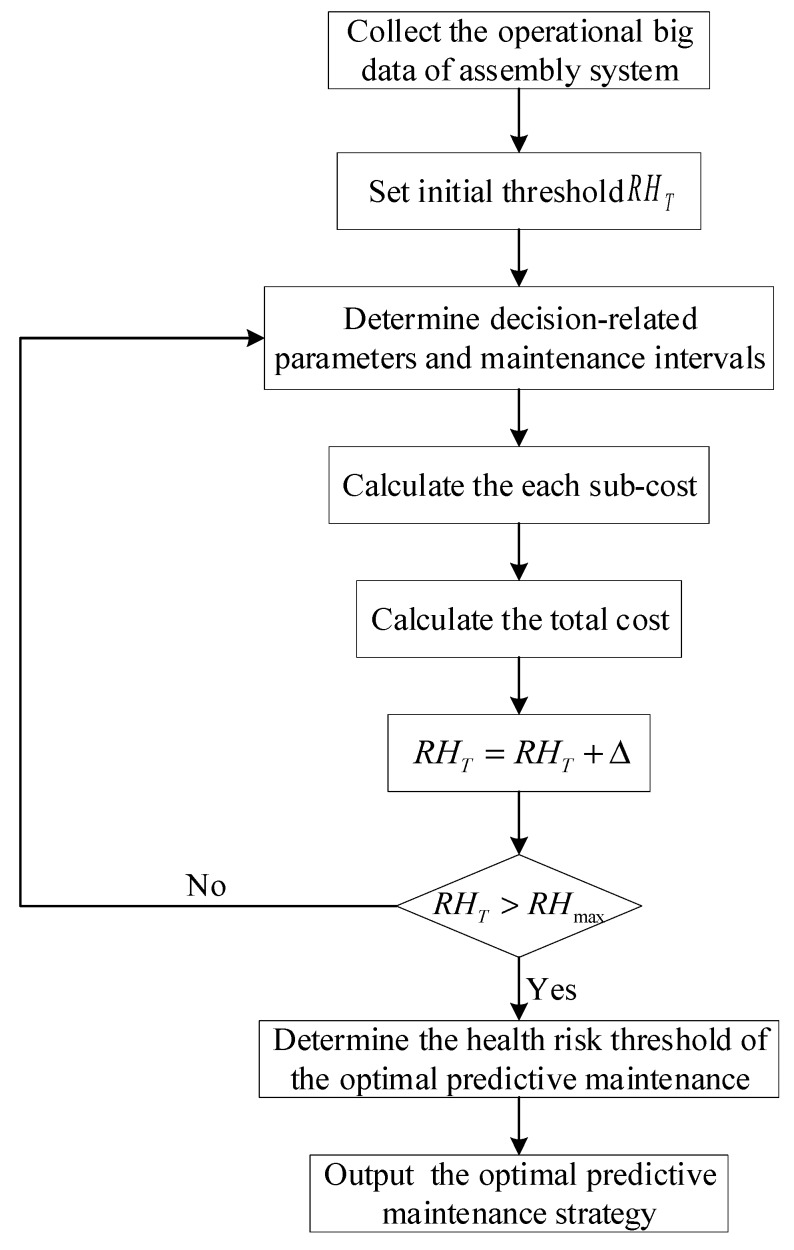
Diagram of health risk-oriented predictive maintenance decision-making mechanism.

As shown in Figure 6, the specific process of equipment predictive maintenance decision-making can be described as follows:

- Step 1: Collecting the operational big data of the assembly system, such as operation, risk, and maintenance data;
- Step 2: Providing an initial health risk threshold $RH_{T}$;
- Step 3: Determining the relevant decision parameters and maintenance intervals based on historical data and expert experience;
- Step 4: Separately calculating the costs of maintenance $c_{M}$, assembly capacity loss $c_{A}$, and product reliability loss $c_{R}$ under the maintenance threshold and then obtaining the total cost C under the threshold;
- Step 5: Setting the search step size Δ, $RH_{T}=RH_{T}+\Delta$;
- Step 6: Determining whether the optimization process should be terminated. If $RH_{T}>RH_{\max}$, then the optimization process is terminated and Step 7 is performed. Otherwise, proceed to Step 5.
- Step 7: Outputting the optimal health risk threshold and the corresponding predictive maintenance strategy.

## 5. Case Study

### 5.1. Background

The enhancement of product features is accompanied by an increase in process complexity and precision. The high-complexity and high-precision assembly process make manufactures have higher and higher reliability requirements for the assembly system. In engineering practice, system defects are often not detected in time, due to the multiple stations of product assembly and complex assembly lines. This condition affects the quality of the assembly process during system operation, resulting in KRC variations, which affect the product reliability, thereby causing quality accidents and immeasurable losses. Taking automotive products as an example, as the heart of automotive products, engines are particularly important for assembly quality. According to statistics, in the warranty period, the quality accidents caused by the decline in the state of engine assembly systems accounted for a large proportion of the total number of product quality accidents. The engine cylinder head is the core and most basic key component of the engine. In addition to supporting the engine frame together with the cylinder block, the engine cylinder head also serves as the reference component for assembling engines. Therefore, once the health state of the assembly system of the engine cylinder head decline, it will cause KRCs dimensional variations of the engine cylinder head during the assembly stage. Moreover, during the actual assembly process, since the cylinder head is the assembly reference component of the engine, its assembly variations can cause the overall engine assembly quality decline, which leads to a decrease in manufacturing reliability of engine and quality accident.

In summary, health management of the assembly system of the engine cylinder head should be conducted to improve the health state. Therefore, during assembly, the assembly system health of the engine cylinder head should be comprehensively analyzed, and maintenance solutions should be proposed to minimize the number of quality accidents. The cross-sectional view and assembly structure of the engine cylinder head are shown as follows.

### 5.2. Numerical Example

The partial assembly system consisting of three assembly devices with equipment numbers 1, 2, and 3 is selected as the case study object in this section. The technical method proposed in this paper is verified.

#### 5.2.1. Health Risk Modeling

• Step 1 Risk identification

**Figure 7:**
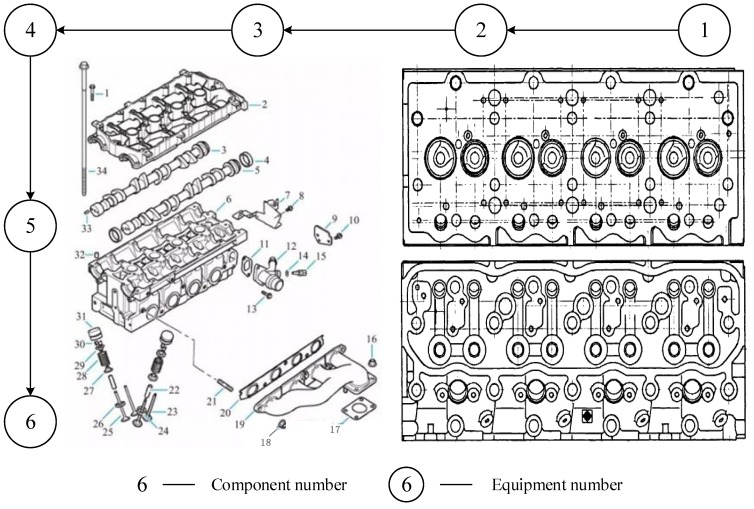
Structure and sectional view of the engine cylinder head assembly.

First, on the basis of the analysis of the design documents of the manufacturer and Figure 7, the structural features of the engine cylinder head can be identified, and the assembly structure information is obtained. Second, based on the obtained structural information, the structural features sensitive to the reliability of the cylinder head are screened out, and the KRS is determined. Third, via the axiomatic domain mapping method, the KRS is gradually mapped from the structure domain to the process domain, and all KRC information of the engine cylinder head is obtained, namely, KRC1, KRC2, and KRC3. Finally, the KRC database of the engine cylinder head is updated in a timely manner. 

• Step 2 Risk quantification

The variation vector represents the variations caused by the degree of freedom of each part. In the assembly process of the engine cylinder head, the number of degrees of freedom is three. In other words, each 2D part has two translational degrees of freedom and one rotational degree of freedom. Therefore, the variation $x_{ikj}$ can be represented as ${\left(\Delta x_{ikj},\Delta y_{ikj},\Delta \theta_{ikj}\right)}^{T}$, where $\Delta x_{ikj}$ and $\Delta y_{ikj}$ represent the variations of the two translational degrees of freedom and $\Delta \theta_{ikj}$ denotes the variation of the rotational degree of freedom. If the *k*th station does not involve the assembly process of the *i*th KRC, then the corresponding term is represented by 0. Then, data of the KRCs process variation $x_{ikj}$ can be obtained by analyzing the operational data of the assembly system. The resulting vectors are taken into Equations (6) and (7) for calculation, and the variation vector of the assembly process of each KRC $\Delta X_{i}$ can be obtained, as follows:

$$\Delta X_{1}={\left(1.53\;1.07\;0.41\right)}^{T},\;\Delta X_{2}={\left(1.49\;0.85\;0.33\right)}^{T},\;\mathrm{and}\;\Delta X_{3}={\left(1.28\;0.91\;0.56\right)}^{T}$$

By using the engineering experience and the fuzzy TOPSIS method, the specific expressions of constant coefficient matrix K and constant D are obtained as follows:

$$K_{1}=(7.21\;6.75\;5.31),\;K_{2}=(4.31\;5.28\;6.73),\;K_{3}=(7.51\;5.17\;6.78)$$

and

D=8.29

These results are brought into Equation (9) for calculation, and the value of L is obtained:

$$L=K_{1}\Delta X_{1}+K_{2}\Delta X_{2}+K_{3}\Delta X_{3}+D=59.96$$

In accordance with the design requirements of the manufacturer, $L_{\max}=70$. Then, the value of k is obtained.

$$k=\frac{L}{L_{\max}}=0.86.$$

The risk parameters table is obtained and shown in Table 1. Based on the value of k, the corresponding health risk cost $C_{RH}$ and the probability of occurrence P in the case can be obtained.

Therefore:

$$C_{RH}=3.95$$

P=0.17

The health risk value is as follows:

RH=59.96×3.95×0.17=40.263

In summary, the value of the assembly system health risk of the engine cylinder head is 40.263.

#### 5.2.2. Predictive Maintenance Decision-Making

Other types of data based on cost data should be collected before making maintenance decisions, as shown in Table 2 for specific parameters and values.

As the value of k differs, the corresponding cost $C_{RH}$ and occurrence probability P of health risk are different. Therefore, according to Table 1, the segmentation basis of the total cost function C can be obtained by calculation, then:

$$C=\{\begin{array}{cc} f_{1}(RH_{T}), & 0<RH_{T}\leq 16.492\\ f_{2}(RH_{T}), & 16.492<RH_{T}\leq 36.960\\ f_{3}(RH_{T}), & 36.960<RH_{T}\leq 47.005 \end{array}$$

**Figure 8:**
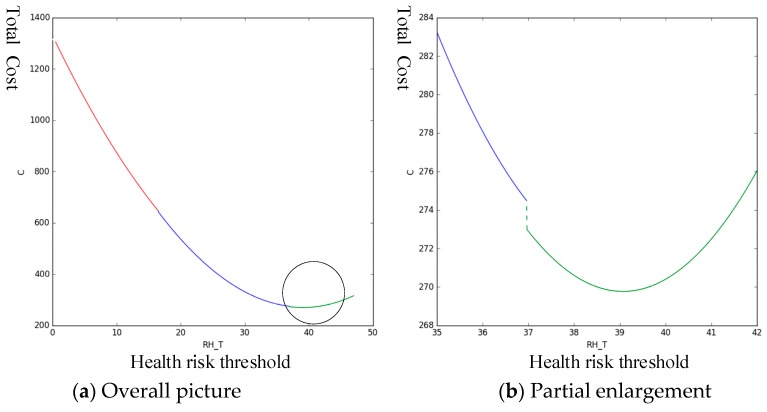
Trend in total costs under different health risk thresholds.

The parameters in Table 2 are taken into the expression of C, and Python is used to analyze the change trend of the total cost C of predictive maintenance under different health risk thresholds, as shown in Figure 8:

In Figure 8, the left graph shows the trend of total cost C over the entire range of (0,47.005]. When the health risk threshold is in the range of [35,42], the trend of C tends to decline initially and then increase. Therefore, a minimum is observed in this interval. As shown in the right graph, the interval is enlarged. The minimum value of C occurs when the health risk threshold is 39.068. Therefore, the optimal predictive maintenance threshold, in this case, is 39.068. Table 3 presents the corresponding predicted maintenance strategy.

In summary, the assembly system of the engine cylinder head is monitored in real time, and when the health risk value reaches 39.068, the maintenance is performed.

### 5.3. Sensitivity Analysis

For the abovementioned maintenance decision model, the values of the parameters will be different in various products and assembly systems. Hence, the predictive maintenance optimization strategy will be different. Therefore, given the effect of this phenomenon on the output of the maintenance decision model and to prove the validity of the proposed method, this section presents the following sensitivity analysis.

Table 4 shows a comparison of the optimal predictive maintenance strategies for different $c_{e}$ values. The result shows that as each maintenance cost increases, the optimal health risk threshold of predictive maintenance increases.

Table 5 shows a comparison of the optimal predictive maintenance strategies for different β values. The result shows that reducing the risk escalation factor can increase the optimal health risk threshold.

Table 6 shows a comparison of the optimal predictive maintenance strategies for different σ values. The result shows that decreasing the cost corresponding to unit reliability loss can increase the optimal health risk threshold.

### 5.4. Comparative Study

In this paper, the reliability risk, cost, and probability of occurrence are comprehensively considered in the calculation of the assembly system health risk, and the health risk is used as the threshold for assessing whether to perform the maintenance activity. However, in the traditional risk quantification model, only the cost and probability of occurrence are considered as risk variables. Therefore, to prove the validity of the proposed model further, a comparative analysis is conducted.

**Figure 9:**
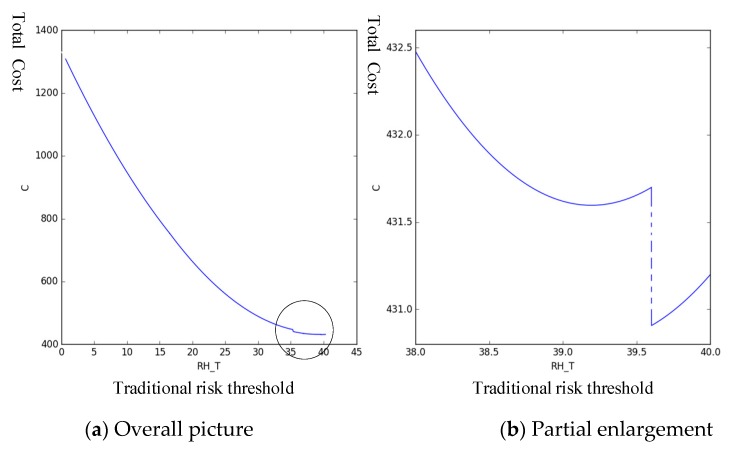
Trend in total costs under new different risk thresholds.

**Figure 10:**
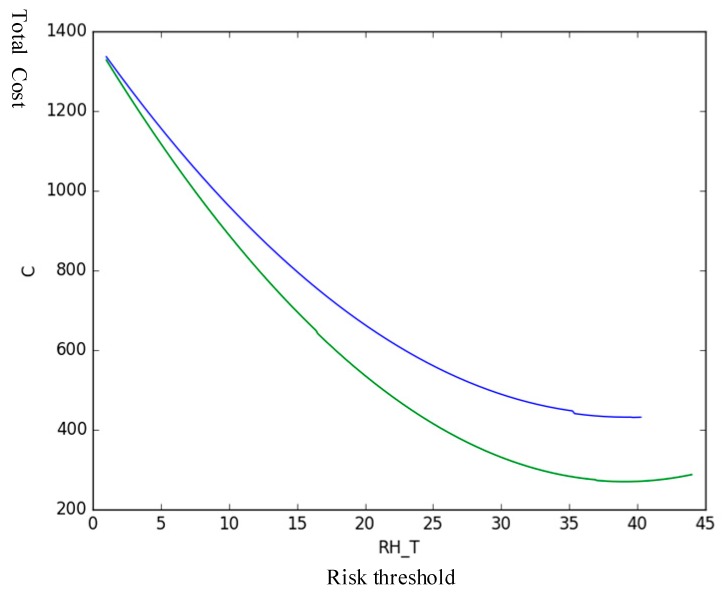
Trends in total costs under two risk thresholds.

The new risk threshold $R_{T}$ is defined with only the risk cost and occurrence probability as variables, that is, the effect of risk on product reliability is considered constant. Other parameters are unchanged. Python software is used to analyze the trend of total cost under different risk thresholds, and the following figure are obtained, as shown in Figure 9 and Figure 10.

Correspondingly, the following table is obtained.

Table 7 shows that the proposed method can save 37.40% of the total costs in maintenance compared with the traditional risk as the maintenance threshold. Therefore, in practical applications, the proposed method can effectively help companies save costs when performing predictive maintenance activities.

## 6. Conclusions

The release of ISO 9001:2015 has lead risk-based thinking to be a hot topic in the field of quality management, and risk-oriented quality management has become a mainstream trend. This study analyzed the health state of product assembly systems and made risk-based maintenance decisions. First, the health state of the product assembly system was analyzed from the perspective of the assembly operation process, and the concept of assembly system health risk was proposed. Second, the quantitative relationship between the process variations of KRCs and the product reliability loss was established, and combined with occurrence probability and cost, the health risk of product assembly system was quantified comprehensively. Third, with the assembly system health risk as the threshold and the minimization of total cost as the goal, the predictive maintenance decision model of the product assembly system was established. Finally, the proposed model was validated by using the engine cylinder head as an example. Analysis results showed that the model is suitable for the health state analysis and predictive maintenance activity decision of the product assembly system, and the proposed model can save 37.40% of the total cost compared with the method of traditional risk as the maintenance threshold.

However, certain deficiencies in the model of this paper, such as not considering specific maintenance methods, still exist. Therefore, in future works, further research can be conducted from the following aspects:

1. The determination of how different maintenance methods affect the assembly system health risks can be further explored.
2. Based on the original decision-making model, the assembly task can be considered to determine the health risk threshold for performing maintenance activities with the minimum total cost.

## Figures and Tables

**Table 1:** Risk parameter values.

Item	k		
	(0,0.4]	(0.4,0.8]	(0.8,1]
$C_{RH}$ (k)	1.90	3.00	3.95
P	0.31	0.22	0.17

**Table 2:** Maintenance parameter values.

Parameter	Value	Parameter	Value
ξ	0.30	$t_{m}$ (day)	0.55
ΔRH	1	β	0.69
$c_{e}$ (k)	2.00	$RH_{\max}$	47.005
δ (k)	0.07	σ (k)	0.90

**Table 3:** Parameter values of the optimal predictive maintenance strategy.

$\mathrm{Health}\;\mathrm{Risk}\;\mathrm{Threshold}\;RH_{T}$	Total Cost C (k)
39.068	269.767

**Table 4:** Optimal predictive maintenance strategy under different $c_{e}$ values.

$c_{e}\;(k)$	$\mathrm{Health}\;\mathrm{Risk}\;\mathrm{Threshold}\;RH_{T}$
2.00	39.068
3.50	46.384
4.00	47.000

**Table 5:** Optimal predictive maintenance strategy under different β values.

β	$\mathrm{Health}\;\mathrm{Risk}\;\mathrm{Threshold}\;RH_{T}$
0.69	39.068
0.55	43.885
0.50	47.005

**Table 6:** Optimal predictive maintenance strategy under different σ values.

σ (k)	$\mathrm{Health}\;\mathrm{Risk}\;\mathrm{Threshold}\;RH_{T}$
0.90	39.068
0.75	40.342
0.50	47.004

**Table 7:** Comparison of the proposed method with the method of traditional risk as maintenance threshold.

Method	Total Cost	Cost Saving Rate
The proposed method	269.767	37.40%
The traditional method	430.907	—
